# Individualised Structural and Functional Frontostriatal Connectivity Mapping in Adult ADHD

**DOI:** 10.1002/hbm.70652

**Published:** 2026-10-01

**Authors:** Tuija Tolonen, Matti Laine, Kimmo Alho, Sami Leppämäki, Pekka Tani, Anniina Koski, Juha Salmi, Timo Roine

**Affiliations:** ^1^ Department of Neuroscience and Biomedical Engineering Aalto University Espoo Finland; ^2^ AMI Centre, Aalto Neuroimaging Aalto University Espoo Finland; ^3^ Department of Psychology University of Helsinki Helsinki Finland; ^4^ Department of Psychology Åbo Akademi University Turku Finland; ^5^ Terveystalo Healthcare Helsinki Finland; ^6^ Department of Psychiatry Helsinki University Hospital Helsinki Finland; ^7^ Unit of Psychology, Faculty of Education and Psychology University of Oulu Oulu Finland; ^8^ School of Smart Solutions, Metropolia University of Applied Sciences Helsinki Finland

**Keywords:** adult ADHD, DW‐MRI, fMRI, functional and structural connectivity, resting state, working memory updating

## Abstract

Abundant research from early neurocognitive models to recent mega‐analyses has highlighted the central role of frontostriatal circuits in attention deficit hyperactivity disorder (ADHD). However, it remains unclear which specific aspects of functionally organised frontostriatal loops are affected by ADHD and how these disruptions differ across individuals in light of the disorder's marked heterogeneity. We investigated frontostriatal connectivity in individualised circuits in 40 adults with ADHD and 36 neurotypical controls using a multimodal dataset that included diffusion‐weighted magnetic resonance imaging (MRI), as well as functional MRI during working memory updating, resting state, and a naturalistic attention task. Compared with the neurotypical controls, the adults with ADHD exhibited reduced structural connectivity strength in a tract originating from left orbital prefrontal areas. However, structural results did not reproduce in control analyses using different thresholds in individual formation of regions of interest. Functional data revealed weaker connectivity in the ADHD group during verbal and visuospatial working memory tasks in circuits connecting the striatum with the dorsolateral prefrontal cortex, motor regions, and orbitofrontal cortex, while no group differences were found during resting state or the naturalistic task. Comparisons with atlas‐based analyses highlighted the precision of individualised connectivity mapping. Together, these findings suggest that frontostriatal functional connectivity aberrancies in ADHD are driven by striatum‐dependent cognitive processes such as working memory, and that precise mapping of frontostriatal pathways can be achieved through individualised connectivity mapping.

## Key Points

- Compared with neurotypical controls, adults with ADHD had weaker structural and functional connectivity in finely parcellated frontostriatal circuits.
- Functional connectivity aberrancies were driven by striatum‐related cognitive processes, such as working memory updating. Structural group differences were small and sensitive to subjective threshold selection.
- Individual‐specific tracts and regions of interest were successfully constructed with white matter tractography.

## Graphical abstract

We identified compromised structural and functional frontostriatal connectivity in adults with attention deficit hyperactivity disorder via individualised connectivity mapping.

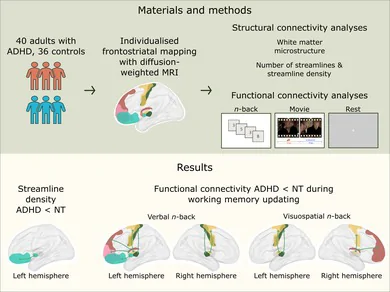

## 1 Introduction

Attention deficit hyperactivity disorder (ADHD) is a neurodevelopmental disorder affecting approximately 8% of the population worldwide (Ayano et al. 2023). In over half of the cases, symptoms of ADHD continue from childhood to adulthood (Faraone et al. 2006). The core symptoms include inattention, which manifests as distractibility and forgetfulness; hyperactivity, often experienced as feeling restless and overenergetic; and impulsivity, commonly shown through impatience and risky behaviours (American Psychiatric Association 2013). Additionally, executive function deficits such as working memory impairments are present in the majority of cases, contributing to core symptoms and adversely impacting daily functioning (Adler et al. 2017; Holst and Thorell 2020; Willcutt et al. 2005).

Adults with ADHD face different challenges in their everyday life compared with children with ADHD as the demands of independent life increase in adulthood, and adults with ADHD often struggle maintaining employment, managing finances, and sustaining long‐term relationships (Kosheleff et al. 2023). While ADHD in childhood has been extensively investigated, adult ADHD and its neural underpinnings are still poorly understood. Given that the ADHD symptom expression changes and the brain continues to develop from childhood to adulthood (Franke et al. 2018; Grayson and Fair 2017; Lebel et al. 2008; Mills et al. 2016), studying adults with ADHD is essential for understanding their specific challenges, elucidating the brain mechanisms underlying symptoms, and developing age‐appropriate diagnostic and treatment approaches.

Of the many brain regions and circuits associated with ADHD (Faraone et al. 2024), aberrancies in frontal and striatal areas are among the most replicated findings (Cortese et al. 2012; Frodl and Skokauskas 2012; Norman et al. 2016; Yu et al. 2023). Already early neurocognitive models of ADHD emphasised the role of frontostriatal circuits, which are thought to underlie deficits for example in executive functioning, response inhibition, and reward processing (Castellanos and Tannock 2002; Krain and Castellanos 2006; Sonuga‐Barke 2002). More recently, large‐scale mega‐analyses involving data from thousands of affected individuals and controls have further underscored the contribution of frontostriatal pathways to ADHD symptoms. In children, such studies have found reduced grey matter volume in striatal and frontal regions, as well as increased resting‐state functional connectivity between them (Hoogman et al. 2017, 2019; Norman et al. 2024). In adults with ADHD, some of the mega‐analyses reported no robust volumetric alterations in striatal or cerebro‐cortical grey matter (Hoogman et al. 2017, 2019). However, a recent mega‐analysis reported reduced frontostriatal white matter volume adjacent to the orbitofrontal cortex (OFC) bilaterally in adults with ADHD (Cupertino et al. 2020). This suggests that connectivity alterations, rather than regional grey matter differences, could be a more reliable marker of frontostriatal dysfunction in adults with ADHD, possibly reflecting the specialisation of frontostriatal functional circuits (Klein et al. 2025) and the general structural and functional brain network maturation from childhood to adulthood (Baker et al. 2015; Grayson and Fair 2017; Park et al. 2022; Uddin et al. 2011).

Frontal cerebro‐cortical regions form reciprocal connections through the striatum to the thalamus and back to the cerebral cortex, giving rise to parallel and partially segregated loops (Alexander et al. 1986; Haber 2016). These circuits are organised along a rostral‐caudal and, to a lesser extent, medial‐lateral gradient (Haber 2016; Jarbo and Verstynen 2015). In this arrangement, anterior and ventral regions of the striatum are more densely connected to orbital and medial prefrontal areas, while posterior and dorsal regions are more strongly connected to lateral prefrontal, premotor, and motor cortices (Alexander et al. 1986; Gordon et al. 2021; Haber 2016; Jarbo and Verstynen 2015; Parkes et al. 2017; Tziortzi et al. 2014). Although their functional territories interact substantially (Haber 2016), distinct frontostriatal circuits have been associated with specific functions. For example, circuits linking the tail of the caudate nucleus to the dorsolateral prefrontal cortex (dlPFC) support executive functions such as working memory, and are especially important in working memory updating, a process in which information held in mind is actively replaced with new relevant content (Baier et al. 2010; O'Reilly and Frank 2006; Rottschy et al. 2012; Wang et al. 2019; Yaple et al. 2019). Connections between the ventral and rostral striatum and orbital and medial prefrontal regions are mostly involved in emotional and motivational processing (Haber 2011), and pathways connecting the posterior putamen to premotor and motor cortices subserve motor planning and execution (Nakano et al. 2000). These distinctive patterns of connectivity suggest that different frontostriatal circuits may contribute in unique ways to ADHD symptomatology. Yet, surprisingly few studies have examined the specific patterns of frontostriatal connectivity in ADHD, particularly in adults.

Structural studies of frontostriatal connectivity, apart from the mega‐analysis introduced above (Cupertino et al. 2020), have mostly observed reduced fractional anisotropy (FA) and increased mean diffusivity in adults with ADHD (Chiang et al. 2022; Konrad et al. 2010; Schweren et al. 2016), although opposite results have also been reported (Chaim et al. 2014). Beyond microstructure, altered structural network organisation has been observed in frontal and striatal areas (Sidlauskaite et al. 2015; Tolonen et al. 2023; Wang et al. 2021). Furthermore, reduced functional connectivity has been observed between the inferior prefrontal cortex (PFC) and caudate nucleus during motor inhibition (Cubillo et al. 2010) and between the anterior cingulate cortex (ACC)/medial PFC and putamen during motivational control (Ceceli et al. 2020). During working memory tasks, both increased and decreased functional connectivity across widespread networks including the caudate nucleus, putamen, and multiple cerebro‐cortical and cerebellar regions has been reported (Tolonen et al. 2024; Wolf et al. 2009). However, in addition to the limited number of studies, prior research has mostly focused either on a small subset of frontostriatal regions or tracts (Ceceli et al. 2020; Chiang et al. 2022; Cubillo et al. 2010), or examined broad whole‐brain patterns (Chaim et al. 2014; Gehricke et al. 2017; Konrad et al. 2010; Sidlauskaite et al. 2015; Tolonen et al. 2023, 2024; Wang et al. 2021; Wolf et al. 2009). A circuit‐specific approach could provide more targeted insights into the relevance of functionally distinct frontostriatal circuits for adult ADHD.

The goal of the present study was to specify the frontostriatal dysconnectivity patterns in adult ADHD by analysing structural and functional connectivity in finely parcellated, individually defined circuits. Our first aim was to reconstruct the architecture of frontostriatal loops at single‐participant level, based on white matter tractography seeded from distinct regions of the striatum to the frontal cerebral cortex. Precise, participant‐specific mapping is essential, as frontostriatal organisation can vary substantially between individuals (Gordon et al. 2021; Tziortzi et al. 2014) and atlas‐based analysis may hence not capture the related group differences accurately. Our second aim was to investigate ADHD‐related alterations within these circuits using multimodal data. We assessed both structural connectivity (via diffusion‐weighted magnetic resonance imaging, DW‐MRI) and functional connectivity during rest, working memory updating tasks, and a naturalistic attention condition (via functional MRI, fMRI).

We employed two *n*‐back working memory tasks, verbal and spatial, to target the updating component of working memory implicated in frontostriatal circuitry, particularly the dlPFC. *n* ‐back tasks are widely used in neuroimaging of working memory updating (Wang et al. 2019; Yaple et al. 2019), and the verbal and spatial variants were included to engage partially dissociable subsystems of working memory. We also included a novel naturalistic sustained attention paradigm shown to detect aberrant activity in adults with ADHD in this sample (Salmi, Metwaly, et al. 2020). We used this paradigm to explore if altered frontostriatal connectivity reflects real‐world cognitive tasks. Including a naturalistic task was motivated by the role of the frontostriatal system in attention and ADHD (Castellanos and Tannock 2002; Cupertino et al. 2020; Meck and Benson 2002; van Schouwenburg and Cools 2009), as well as its ability to capture brain‐behaviour relationships (Finn 2021), while recognising that no direct evidence demonstrating altered activity in this network during naturalistic tasks was available. Finally, resting‐state fMRI was acquired because of convergent evidence of frontostriatal connectivity alterations in children with ADHD (Norman et al. 2024). This comprehensive approach allowed us to explore whether structural and functional alterations align within the same frontostriatal circuits and uncover how these functional changes are influenced by different behavioural contexts.

Our hypotheses were: (i) Adults with ADHD have fewer white matter streamlines than neurotypical (NT) controls between striatal and frontal cerebro‐cortical regions (see Tolonen et al. 2023); and (ii) adults with ADHD compared with NT adults have weaker working memory related connectivity between frontal and striatal regions (see Tolonen et al. 2024) that are observed at least in the circuits connecting the dorsal caudate and dlPFC associated with executive functions (Baier et al. 2010; Leh et al. 2010; O'Reilly and Frank 2006). Furthermore, we explored group differences also in the white matter microstructure (FA, axial diffusivity (AD) and radial diffusivity (RD)) and in functional connectivity during the naturalistic attention task and resting state but did not set more specific hypotheses about these differences due to the limited or inconsistent previous evidence.

## 2 Materials and Methods

### 2.1 Participants

Forty individuals with ADHD and 36 NT controls took part in this study. Adults with ADHD were recruited by psychiatrists at clinics in the Helsinki metropolitan area (the Neuropsychiatry outpatient clinic of the Helsinki University Hospital; Diacor Healthcare Services, Helsinki, Finland; and ProNeuron, Espoo, Finland) and were pre‐screened in the clinic. NT adults were recruited via e‐mail lists of universities, adult high schools, polytechnics, and vocational schools, and via the personal contacts of the authors. To be accepted to the study, the participants had to be native Finnish speakers, have normal hearing, normal or corrected‐to‐normal vision, cognitive abilities at or above average level, and no contraindications to MRI. Participants were excluded if they had moderate or major psychiatric or neurological conditions (apart from ADHD in the ADHD group), such as learning disorders, depression or anxiety disorders, tic disorder, or past head trauma that had demanded treatment. The study was conducted according to the Declaration of Helsinki. All participants gave written informed consent to participate in the study. The study was reviewed and approved by the Ethics Committee for Gynaecology and Obstetrics, Pediatrics and Psychiatry of the Helsinki University Hospital. The participants were compensated by 60 € if they participated only to the parts described in the present study, and by 240 € if they continued to a working memory training trial described elsewhere (Salmi, Soveri, et al. 2020).

ADHD diagnoses were made by experienced psychiatrists based on the Diagnostic and Statistical Manual of Mental Disorders, Fourth Edition (DSM‐IV; American Psychiatric Association 1994). All participants with ADHD met criteria for both inattention and hyperactivity/impulsivity or only for inattention, according to the Conners' Adult ADHD Diagnostic Interview for DSM‐IV (Epstein et al. 2001). Two of the participants had hypothyroidism, four had migraines, and two had experienced mild symptoms of epilepsy in childhood requiring no later treatment. The participants with hypothyroidism and migraine had prescription medication, and one participant had a selective serotonin re‐uptake inhibitor prescription for mild depression. In addition, 33 participants with ADHD were using stimulants but were instructed to have a 24‐h wash‐out period before the MRI session.

### 2.2 Neuropsychological Assessment and Self‐Ratings

General cognitive abilities were assessed with two tasks (Matrix Reasoning and Vocabulary) from the Wechsler's Adult Intelligence Scale III (WAIS‐III; Wechsler 2005). Working memory was assessed with the Digit Span test from WAIS‐III (Wechsler 2005) and with spatial and verbal running memory tasks (Pollack et al. 1959; see Salmi, Soveri, et al. 2020 for full details). For self‐assessment of ADHD symptoms, we used the Adult ADHD Self‐Report Scale (ASRS; Kessler et al. 2005). Depression symptoms were rated with the Depression Scale (DEPS; Salokangas et al. 1995), and alcohol use with the Alcohol Use Disorders Identification Test—Consumption (AUDIT‐C; Bush et al. 1998).

### 2.3 MRI Acquisition and Preprocessing

#### 2.3.1 Imaging Acquisition

All images were collected at Advanced Magnetic Imaging Centre (Aalto University, Espoo, Finland) with a Siemens MAGNETOM Skyra 3 T scanner (Siemens Healthcare, Erlangen, Germany) mounted with a 30‐channel head coil. We collected structural MRI with a T1‐weighted MPRAGE sequence (repetition time (TR) 2.5 s, field of view (FOV) 256 × 256, 1 mm × 1 mm × 1 mm resolution, 176 sagittal slices in total), fMRI images with a gradient‐echo echo‐planar imaging sequence (TR 1.9 s, FOV 64 × 64, 3.1 mm × 3.1 mm × 3.0 mm resolution, 33 axial slices in total), and diffusion‐weighted images with echo‐planar imaging sequence in 64 different gradient directions (*b* = 1000 s/mm^2^) with additional 10 unweighted scans (*b* = 0 s/mm^2^; TR 9 s, 2.5 mm × 2.5 mm × 2.5 mm resolution, FOV 240 × 240, 70 axial slices in total). Tasks were presented with a 3‐micromirror data projector (Christie X3, Christie Digital Systems, Mönchengladbach, Germany) on a semi‐transparent screen which was placed behind the participant's head. The participants had a 24 cm binocular field of view and approximately 34 cm distance to the screen via a mirror located above the eyes.

#### 2.3.2 Preprocessing and Denoising

Full preprocessing details of the MRI data are reported in previous articles (Tolonen et al. 2023, 2024). Briefly, the fMRI preprocessing was conducted using the standard pipeline of fMRIPrep, version 22.1.0 (Esteban et al. 2019; RRID:SCR_016216). T1‐weighted images were corrected for intensity non‐uniformity, skull‐stripped and segmented to cerebrospinal fluid (CSF), white matter (WM), and grey matter. Brain surfaces were reconstructed with FreeSurfer's recon‐all (Dale et al. 1999; RRID:SCR_001847). Functional MRI data were slice‐time corrected, resampled onto their original native space by applying the transforms to correct for head motion, and finally co‐registered to the T1‐weighted image. After fMRIPrep preprocessing, fMRI data were further denoised by using a discrete cosine high‐pass filter with a 128 s cut‐off, regressing out volumes identified as motion outliers (framewise displacement > 0.5 mm or standardised DVARS > 1.5), and regression of the CSF and WM global signals and six head motion parameters with their temporal derivatives and quadratic terms (4 CSF global signals, 4 WM global signals, and 24 head motion parameters in total; Satterthwaite et al. 2013). DW‐MRI data were corrected for eddy current and susceptibility induced distortions and for participant motion (Andersson and Sotiropoulos 2016; Irfanoglu et al. 2012).

### 2.4 fMRI Conditions

#### 2.4.1 *n*‐Back Tasks

*n* ‐back tasks are working memory updating tasks, in which a continuous series of stimuli is presented to the participant (Kirchner 1958). Their task is to match the currently presented stimulus with the one presented *n* items before. For example, in a 3‐back task, the participant's task is to decide for each stimulus whether it is the same as the one presented three items before. In the present study, the participants conducted two types of single *n*‐back tasks during fMRI, one at a time. In the visuospatial *n*‐back task, they were presented with white squares in eight outer locations of a 3 × 3 matrix with a fixation point in the centre, and the participant's task was to indicate whether the current white square occurred in the same location as the square *n* trials before. In the digit *n*‐back task, numbers from 1 to 9 were displayed at the centre of the screen and the participant's task was to indicate whether the current number was the same as the number *n* trials before. The location/digit was shown for 1500 ms and the interstimulus interval was 450 ms. There were four different *n*‐back levels (0‐, 1‐, 2‐, and 3‐back), divided into separate blocks and presented in a counterbalanced order (0‐back, 1‐back, 2‐back, 3‐back, 3‐back, 2‐back, 1‐back, 0‐back, etc.). There were 10 trials in each block, and on average, two of them were randomly match trials (targets). The participants were instructed to press a match button for match trials and a non‐match button for non‐match trials, except for the 0‐back where they only pressed the non‐match button for all stimuli. The current *n*‐back level was instructed at the beginning of each block. All task levels were presented five times in total, and each task lasted approximately 13 min.

#### 2.4.2 Resting State

In the resting‐state condition, the participants were instructed to look at a white cross presented in the centre of a grey screen. The acquisition lasted approximately 7 min.

#### 2.4.3 Naturalistic Attention Task

During the naturalistic attention condition, participants watched a 14 min 30 s film excerpt with audio, showing a socially rich interaction. The clip was taken from the Finnish film *Three Wise Men* (2008, directed by Mika Kaurismäki), in which three former friends unexpectedly reunite, go to a pub, and engage in animated conversation. Most of the scenes were shot from a steady camera angle, typically displaying all three characters interacting at once. We superimposed intermittent auditory distractors, each lasting 15 s, onto the original soundtrack: A speech stimulus, which was Ronald Reagan's First Inaugural Address (June 17, 1980; see www.reaganlibrary.gov); a musical piece, which was *The Believer* by John Coltrane (1964, Prestige Records); and white noise. In addition, there were 15 s distractor‐free periods. The order of the three distractor types and distractor‐free periods was pseudo‐randomised so that the same type did not repeat consecutively. The sound level of distractors was adjusted subjectively in pilot testing to be comparable to the film dialogue (about 85 dB SPL at the eardrum) and set so that the film could still be followed, but only with increased effort. Participants were instructed to focus on the film while disregarding the irrelevant sounds. To ascertain that the participants attended to the film, they were told that a quiz would follow the viewing after the MRI session. The quiz contained 36 three‐alternative questions spanning the entire excerpt, addressing both story details and visual context, and responses were scored as correct or incorrect. The audio was delivered through an UNIDES ADU2a system (Unides Design, Helsinki, Finland) using plastic tubes and porous EAR‐tip earplugs (Etymotic Research, ER3, IL, USA).

### 2.5 Defining Regions of Interest

The striatum was divided into regions of interest (ROIs) based on a parcellation by Janssen et al. (2015) (Figure 1). The caudate nucleus, putamen, and ventral striatum were separated to six regions on both hemispheres: dorsal caudate nucleus, ventral caudate nucleus, rostral caudate nucleus and nucleus accumbens, rostral putamen, dorsal putamen, and caudal putamen. Similar clustering of the striatum based on its functional connectivity has also been reported in other studies (e.g., Jung et al. 2014; Liu et al. 2021). Striatal ROIs were transformed to the participants' DW‐MRI space for tractography and DW‐MRI analyses, or to T1‐weighted space for fMRI analyses with ANTs' symmetric image normalisation (SyN; Avants et al. 2008; RRID:SCR_004757).

**FIGURE 1:**
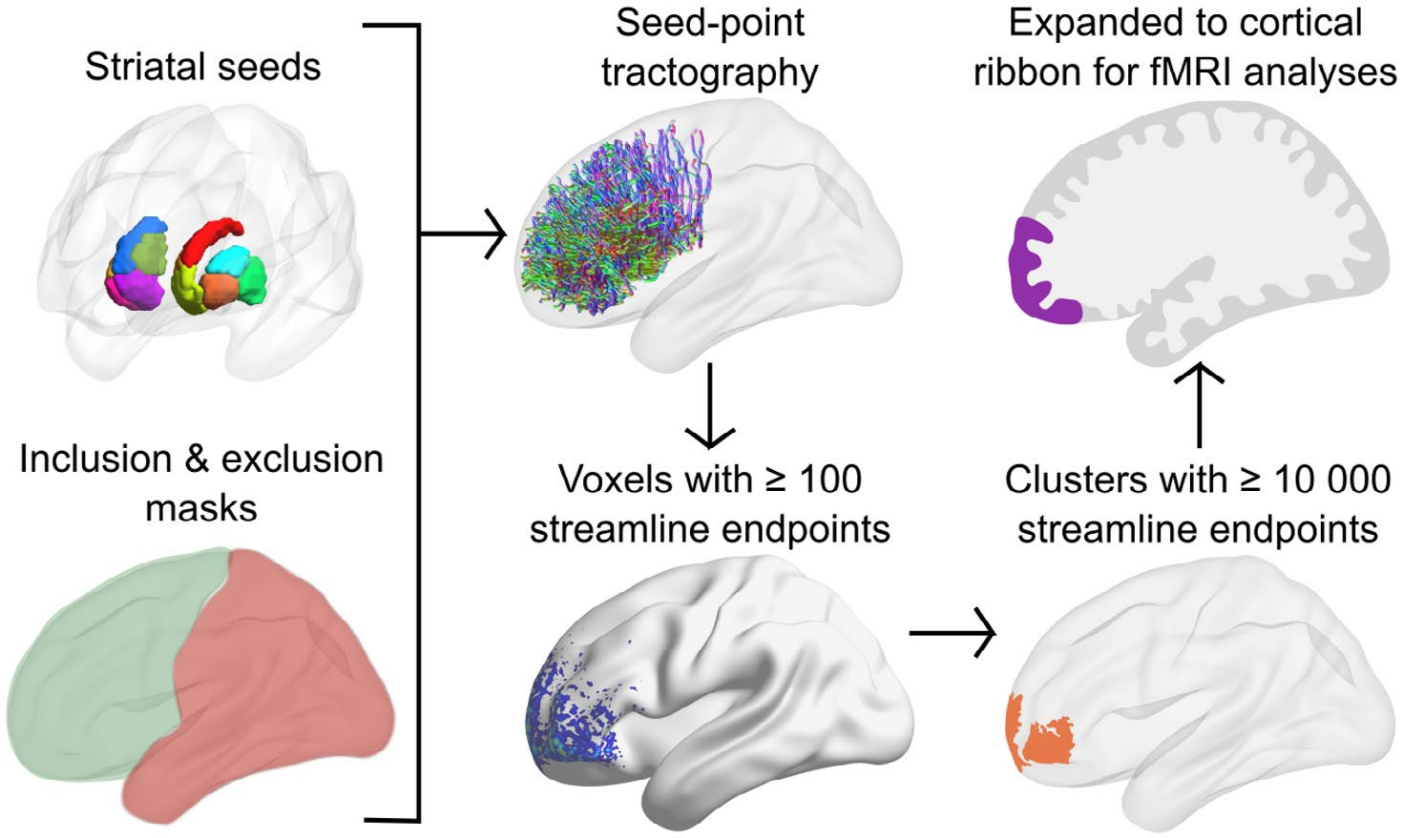
Schematic overview of cerebro‐cortical ROI formation. One million streamlines were generated from each striatal seed using predefined inclusion and exclusion masks. For each seed‐point, voxels containing at least 100 streamline endpoints were then identified. Voxel clusters comprising a minimum of 10,000 streamline endpoints were selected for cerebro‐cortical ROIs. For fMRI analyses, these ROIs were expanded to encompass the entire cortical ribbon. See main text for further details.

A cerebro‐cortical ROI pair for each striatal region was then defined by selecting areas that were most densely connected to it based on the number of white matter streamlines. Specifically, first a seed‐based, anatomically‐constrained tractography using constrained spherical deconvolution (CSD) was conducted from each striatal ROI using MRtrix3 (Smith et al. 2012; Tournier et al. 2007, 2019; RRID:SCR_024123). One million streamlines were created from each striatal seed ROI. Tractography was constrained to the frontal lobe unilaterally with individual participant‐specific inclusion and exclusion masks. The inclusion masks included all areas of the frontal cortex from the Destrieux atlas (Destrieux et al. 2010). The exclusion masks covered the rest of the brain (temporal, parietal, occipital, and insular cerebral cortices and the cerebellum), including subcortical structures (the thalamus, pallidum, hippocampus, and amygdala), derived with FSL's FIRST algorithm (Patenaude et al. 2011; Smith et al. 2004; RRID:SCR_002823), and the mid‐sagittal plane. After tractography, a streamline endpoint map was created for each seed‐point with MRtrix3's tckmap‐ends_only, and cerebro‐cortical voxels that included less than 100 streamline endpoints were excluded. Then, neighbouring voxels, either by the voxel face, edge, or vertex, were defined as clusters with MRtrix3's maskfilter‐connectivity, and only clusters including at least 10,000 streamline endpoints in total were selected. The selected clusters formed cerebro‐cortical ROIs and were used as is for DW‐MRI analyses and expanded to cover the whole cortical ribbon for the fMRI analyses with FreeSurfer's surf2vol. See Figure 1 for a schematic overview of the cerebro‐cortical ROI formation.

These voxel‐ and cluster‐wise thresholds were chosen by visually inspecting the cerebro‐cortical endpoints and by selecting thresholds that yielded ROIs which fitted best to known frontostriatal models. Visual inspection was done with 18 participants from the NT group and validated with the rest of the NT group. However, as the threshold selection was based on subjective decisions, the analyses were repeated with several thresholds to ensure the robustness of the findings. Complementary analyses were conducted with voxelwise thresholds of 75 and 125, and a clusterwise threshold of 7500.

To further evaluate the robustness of individual tract reconstructions, we compared them to tracts generated with pre‐defined cerebro‐cortical regions. Connection density (defined as the number of streamlines divided by the mean number of voxels in the striatal and cortical ROIs; see Section 2.7) was used as an index of how accurately each striatal ROI was linked to its cerebro‐cortical counterpart. The pre‐defined cerebro‐cortical ROIs were selected from the Schaefer 200 parcellation (Schaefer et al. 2018) based on prior literature (Alexander et al. 1986; Gordon et al. 2021; Haber 2016; Jung et al. 2014) and used as cortical inclusion masks. The cerebro‐cortical ROIs were clipped with a whole‐brain endpoint mask to get a corresponding voxel count compared with the individually defined ROIs. The chosen regions are illustrated in Figure S1.

### 2.6 Excluded Participants

Some participants were excluded from further analyses during the data processing steps. From resting‐state analyses, two participants from the ADHD group were removed due to inaccurate image field placement during fMRI. Furthermore, because the resting‐state scan was shorter than the other fMRI paradigms, we applied a conservative criterion for the proportion of motion outliers to ensure sufficient data for reliable functional connectivity estimation (Van Dijk et al. 2010). Participants with more than 30% of motion outlier volumes during the resting‐state acquisition were excluded. This resulted in the removal of three additional participants from the ADHD group and two from the NT group. In the longer *n*‐back and naturalistic attention paradigms, no participants were removed since the number of volumes remained sufficient after motion outlier removal. The proportion of motion outlier volumes was also within reasonable limits and did not exceed 40% in any participant in these tasks. The final sample size was 40 ADHD and 36 NT participants for the *n*‐back tasks and for the naturalistic attention condition, and 35 ADHD and 34 NT participants for the resting state condition. Furthermore, all cerebro‐cortical ROIs were not reconstructed (i.e., no clusters containing enough streamline endpoints emerged) in one participant from the NT group. The participant is included in analyses with successful ROI formation (bilateral dorsal caudate circuits and left‐sided caudal, dorsal and rostral putamen circuits). Additional analyses where the participant was removed were conducted as a control. In these analyses, the results remained unchanged.

### 2.7 Functional and Structural Connectivity Measures

To estimate structural connectivity, whole‐brain CSD‐based anatomically‐constrained tractography was first performed in MRtrix3 with 10 million streamlines seeded from the grey matter—white matter interface (Smith et al. 2012; Tournier et al. 2007). Streamlines were further weighted to correct their density distributions (Smith et al. 2013, 2015). The streamlines connecting each cortical‐striatal ROI pair were extracted from the weighted whole‐brain tractograms by using the ROI inclusion masks (see Section 2.5). The number of extracted streamlines was used as a measure of structural connectivity. Furthermore, because the size of the cerebro‐cortical ROIs could potentially vary substantially between participants due to the definition method, we also measured the streamline density by dividing the number of streamlines by the mean number of voxels in the cortical and striatal ROI.

For functional connectivity, the Nilearn toolbox (Abraham et al. 2014 RRID:SCR_001362) was used to measure Pearson's correlation between the time‐series of each cortico‐striatal ROI pair. Correlation values were Fisher transformed before any further analyses.

### 2.8 Microstructural Analyses

White matter microstructure was analysed using three conventional measures: FA, AD, and RD. Diffusion tensors were first estimated from the preprocessed DW‐MRI data using weighted least‐squares with MRtrix3's dwi2tensor (Basser et al. 1994; Veraart et al. 2013). Mean FA, AD, and RD were then computed from the tensors along the streamlines extracted between each cortico‐striatal ROI pair (see Section 2.7).

### 2.9 Spatial Overlap of Structural and Functional Connectivity

We quantified spatial overlap between structural and functional connectivity using the Dice similarity coefficient. For each striatal seed, we first generated binary top 25% masks within a frontal cortical mask, retaining voxels that exceeded the 75th percentile of the connectivity strengths. Dice coefficients were then computed between the structural and functional top 25% masks for each task × seed combination as Dice = 2|*A* ∩ *B*|/(|*A*| + |*B*|).

### 2.10 Medication Effects on Structural and Functional Connectivity

We evaluated medication effects on functional and structural connectivity using ordinary least squares regression. Analyses were conducted within the ADHD group, because medication was only present in the participants with ADHD. For overall stimulant use, we fit *y* ~ medication use (stimulant users *n* = 33, non‐users *n* = 7) separately for each connectivity outcome. To examine specific medication types, we modelled *y* ~ medication group (methylphenidate users *n* = 29, (lis)dexamfetamine users *n* = 4, non‐users *n* = 7) with reference “non‐user”, yielding the contrasts methylphenidate versus non‐user and (lis)dexamfetamine versus non‐user. We used heteroskedasticity‐robust standard errors (HC3) to account for unequal group sizes.

### 2.11 Statistical Analyses

Normality of the variables was assessed through visual inspection of Q–Q plots and the D'Agostino–Pearson test. For variables in streamline connectivity, streamline density, and microstructure, clear outliers were identified and data points exceeding ±3 standard deviations from the group mean were removed. Between‐group differences in functional connectivity, number of streamlines, and streamline density were tested with independent‐samples *t*‐tests. Analyses with pre‐set hypotheses were conducted with one‐tailed tests and experimental analyses with two‐tailed tests. Overall microstructural properties were examined with one‐way multivariate analysis of variance (MANOVA), examining the impact of group on the multivariate pattern of FA, AD, and RD. For MANOVA results showing significant group effects, post hoc independent‐samples *t*‐tests were performed on each of the three microstructural measures. The relationship between post‐quiz performance and functional connectivity during the naturalistic attention paradigm was examined with Pearson's *r*. Correction for multiple comparisons was performed using the false discovery rate (FDR), applied across the 12 frontostriatal ROI pairs or across the three microstructural measures in the MANOVA post hoc analyses. Because some variables deviated from normality and failed the D'Agostino–Pearson test, all *t*‐tests were re‐run using the nonparametric Mann–Whitney *U*‐test. The pattern of results remained unchanged.

## 3 Results

### 3.1 Background Variables

The ADHD and NT groups did not statistically differ in age, gender, handedness, education level, general cognitive abilities, alcohol consumption, mood, or in quiz performance after the naturalistic attention task. The ADHD group had more inattention and hyperactivity symptoms than the NT control group and performed worse in a spatial running memory working memory task. During the *n*‐back tasks, the participants made more errors when the working memory load increased, but there were no statistically significant group differences in hit rates at any load level. The ADHD participants had faster reaction times in all *n*‐back levels during the visuospatial task, and in the 2‐back level during the digit task. See Table S1 for statistics related to background variables in this sample (also Tolonen et al. 2023).

### 3.2 Reconstructed Frontostriatal Circuits

Cerebro‐cortical ROIs defined with tractography from the six striatal seed points in each hemisphere followed well the patterns described in previous literature (Figure 2). More specifically, in the putamen, the caudal part was mainly connected to primary motor areas, dorsal area to premotor areas and rostral part to OFC. In the caudate nucleus, the dorsal section was primarily connected to the lateral PFC, mostly its dorsal and ventral areas and extending to the frontal pole. The cerebro‐cortical region also covered caudal and dorsal medial areas. The cerebro‐cortical counterpart of the ventral caudate nucleus overlapped largely with the one of the dorsal caudate nucleus but was centred slightly more ventral and rostral, and included more ventromedial PFC areas. The rostral caudate nucleus and nucleus accumbens were predominantly connected to the ACC and orbitofrontal areas, overlapping with the rostral putamen connectivity pattern. There were clear individual differences in the specific cortical endpoint regions, but overall, they followed a similar pattern across participants and across both groups, although the ADHD group showed slightly more inter‐individual variability (Figure 2). Tracts constructed individually for each participant included more streamlines relative to ROI size than the tracts formed by tracking to pre‐selected cortical areas (all *p* < 0.001, see Figure S2). Cortical ROI maps for each seed point including all participants and transformed to MNI‐space are available as Supporting Information of this article.

**FIGURE 2:**
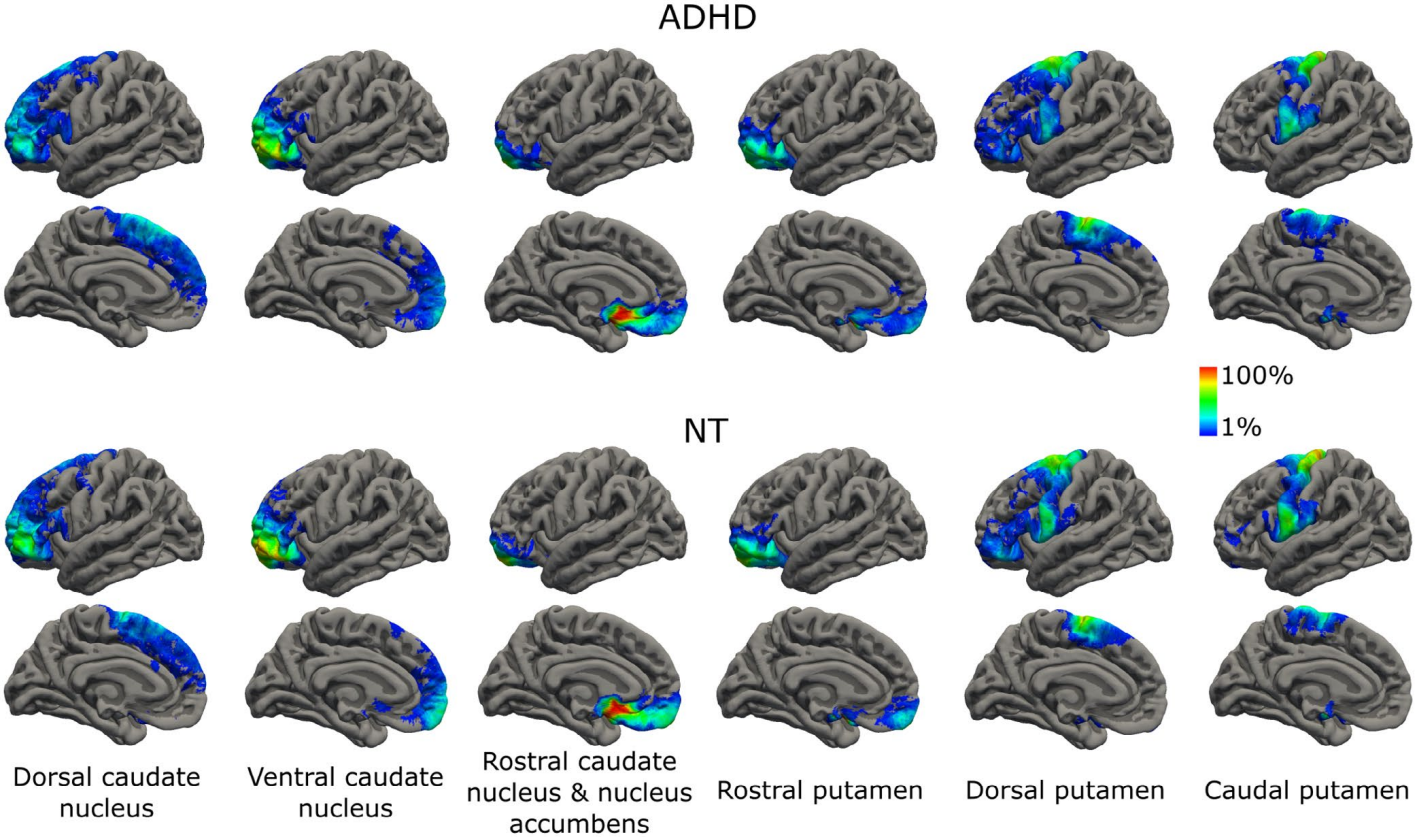
Overlap of cerebro‐cortical ROI locations across individuals within each group. Cooler colours indicate lower overlap, and warmer colours indicate higher overlap. Percent overlap is shown separately for each group.

### 3.3 White Matter Group Differences

#### 3.3.1 Number of Streamlines

There were no group differences surviving FDR‐correction in the number of streamlines connecting any of the frontostriatal ROI pairs. See Figure S3 for further statistics.

#### 3.3.2 Streamline Density

Compared with the NT controls, the adults with ADHD had a significantly decreased streamline density (i.e., fewer streamlines per voxel) between the left rostral putamen and its frontal counterpart (*t*(71) = −2.80, *p* = 0.003; significant after FDR‐correction). See Figure S4 for statistics concerning the other effects.

Group (ADHD, NT) × hemisphere (left, right) interactions were analysed with two‐way ANOVA to evaluate hemispheric lateralisation effects across groups. There were no interaction effects surviving FDR correction (all *p* > 0.30).

#### 3.3.3 Microstructure

No statistically significant differences after FDR‐correction were observed between the ADHD and NT groups in overall microstructural properties in any frontostriatal ROI pair. See Table S2 for statistics.

### 3.4 Functional Connectivity

#### 3.4.1 Digit *n*‐Back

The adults with ADHD had a significantly decreased functional connectivity compared with the NT group during the verbal *n*‐back task in the circuits related to the left dorsal caudate nucleus (*t*(74) = −2.06, *p* = 0.021), ventral caudate nucleus (*t*(73) = −2.20, *p* = 0.015), and rostral putamen (*t*(74) = −2.21, *p* = 0.015), and bilateral caudal (left: *t*(74) = −2.98, *p* = 0.002; right: *t*(73) = −3.02, *p* = 0.002) and dorsal putamen (left: *t*(74) = −3.08, *p* = 0.001; right: *t*(73) = −2.52, *p* = 0.007), all surviving FDR‐correction. See Figure S5 for statistics regarding other functional connectivity analyses for the digit *n*‐back.

#### 3.4.2 Visuospatial *n*‐Back

Compared with the NT adults, the adults with ADHD had a significantly decreased functional connectivity during the visuospatial *n*‐back task in circuits related to the right dorsal caudate nucleus (*t*(74) = −2.43, *p* = 0.009), left caudal putamen (*t*(74) = −2.75, *p* = 0.004), and bilateral dorsal putamen (left: *t*(74) = −3.24, *p* < 0.001; right: *t*(73) = −2.53, *p* = 0.007), all surviving FDR‐correction. Statistics for the other frontostriatal ROI pairs can be found in Figure S6.

Motivated by group differences in out‐of‐scanner spatial running memory performance, we examined associations between visuospatial *n*‐back task‐evoked functional connectivity and spatial running memory performance with Pearson's *r* separately in both groups. Analyses were limited to right‐hemisphere circuits showing group effects, given the right‐hemispheric dominance of spatial working memory (Nagel et al. 2013; Walter et al. 2003). There were no statistically significant correlations in either group (all *p* > 0.26).

To assess whether *n‐*back modality differentially modulates hemispheric functional connectivity across groups, a three‐way ANOVA was performed with factors group (ADHD, NT), task (digit *n*‐back, visuospatial *n*‐back), and hemisphere (left, right). No group × task × hemisphere interactions were statistically significant in any circuit after FDR correction (all *p* > 0.09).

#### 3.4.3 Resting State

No differences between the ADHD and NT groups were observed during the resting‐state condition in any frontostriatal ROI pair (all *p* > 0.05; see Figure S7).

#### 3.4.4 Naturalistic Attention Task

There were no functional connectivity differences between the ADHD and NT groups during the naturalistic attention task in any frontostriatal ROI pair (all *p* > 0.05; see Figure S8). Excluding one participant from the NT group with very low post‐quiz performance (1 point) as a control analysis did not affect the results (all *p* > 0.13). Post‐quiz performance did not correlate statistically significantly with functional connectivity in any circuit (all *p* > 0.065).

#### 3.4.5 Interaction Effects Between Group and Task

To test for possible task specificity of the functional connectivity alterations between groups, group (ADHD, NT) × task (digit *n*‐back, visuospatial *n*‐back, resting state, naturalistic attention task) interaction effects were analysed with two‐way ANOVA. There was a statistically significant interaction between the effects of group and task on functional connectivity in bilateral dorsal putamen circuits (left: *F* (3, 201) = 4.06, *p* = 0.008, right: *F* (3, 201) = 4.99, *p* = 0.002) and right caudal putamen circuit (*F* (3, 201) = 4.31, *p* = 0.006), all surviving FDR correction. Simple main effect analysis showed that the ADHD group had decreased functional connectivity during the digit *n*‐back in bilateral dorsal putamen (left: *t*(67) = −2.71, *p* = 0.008; right: *t*(67) = −2.52, *p* = 0.014) and right caudal putamen (*t*(67) = −2.94, *p* = 0.005) circuits, and during spatial *n*‐back in bilateral dorsal putamen (left: *t*(67) = −3.24, *p* = 0.002; right: *t*(67) = −2.44, *p* = 0.017) circuits. Please note that the statistics differ from the main results in Sections 3.4.1 and 3.4.2 due to participant exclusions in the resting state condition. There were no group differences in resting state or naturalistic attention paradigms in these circuits (all *p* > 0.56).

### 3.5 Spatial Overlap of Structural and Functional Connectivity

Mean dice coefficients between structural and functional connectivity varied between 0.16 and 0.24, depending on striatal seed point and task. Largest dice coefficients were observed for the dorsal and ventral caudate seeds, and the smallest for rostral caudate and putamen seeds. See Table S3 for further statistics.

### 3.6 Complementary Analyses

#### 3.6.1 Effects of Threshold Choice in ROI Formation

Complementary analyses using voxelwise thresholds of 75 and 125 and clusterwise threshold of 7500 yielded partly similar results to the main analyses using thresholds 100 and 10,000. Microstructural results were reproduced (i.e., no group differences emerged) with voxelwise thresholds 75 and 100, but with threshold 125 group differences were found in tracts seeded from rostral caudate nucleus and nucleus accumbens bilaterally. Post hoc analyses revealed that the adults with ADHD, compared with the NT group, had higher RD in the left‐sided circuit and higher AD in the right‐sided circuit. There were no results surviving FDR‐correction in the number of streamlines or streamline density. Functional connectivity analyses were largely repeated with all thresholds: No significant group differences were found for the naturalistic attention or resting‐state conditions, and the ADHD group had reduced connectivity compared with the NT group during both *n*‐back tasks. There were minor differences in the specific circuits depending on thresholds. See section Complementary Analyses in Supporting Information for detailed description of complementary analyses results.

#### 3.6.2 Medication Effects

During the verbal *n*‐back task, overall stimulant use predicted lower functional connectivity in right caudal putamen circuits (stimulant use vs. non‐users: *β* = −0.11, *t*(38) = −2.90, *p* = 0.004). This effect was specific to methylphenidate use (methylphenidate vs. non‐users: *β* = −0.13, *t*(37) = −3.28, *p* = 0.001). In structural connectivity, (lis)dexamfetamine users exhibited a greater number of streamlines than non‐users in the left rostral caudate circuit ((lis)dexamfetamine vs. non‐users: *β* = 4480, *t*(37) = 3.74, *p* < 0.001). No other stimulant use effects on functional or structural connectivity survived FDR correction.

## 4 Discussion

The current work presents a comprehensive investigation of functional and structural alterations within a detailed individualised parcellation of frontostriatal circuits in adult ADHD (see Figure 3 for an overview of the results). Using DW‐MRI‐based tractography, we were able to define the mosaic of parallel, functionally organised frontostriatal pathways on an individual level, following the rostral‐caudal gradient commonly observed in humans (Gordon et al. 2021; Jarbo and Verstynen 2015; Parkes et al. 2017; Tziortzi et al. 2014) and other primates (Alexander et al. 1986; Haber 2016). Furthermore, in line with the first hypothesis, lower streamline density was observed in the ADHD group than the NT group. This group difference was observed in orbitofrontal pathways. Moreover, consistent with the second hypothesis, we found reduced functional connectivity in ADHD during working memory updating. These effects were observed between dorsolateral, orbital, motor, and premotor cortical regions and associated striatal parts. Yet, no group differences during resting state or the naturalistic attention task were found.

**FIGURE 3:**
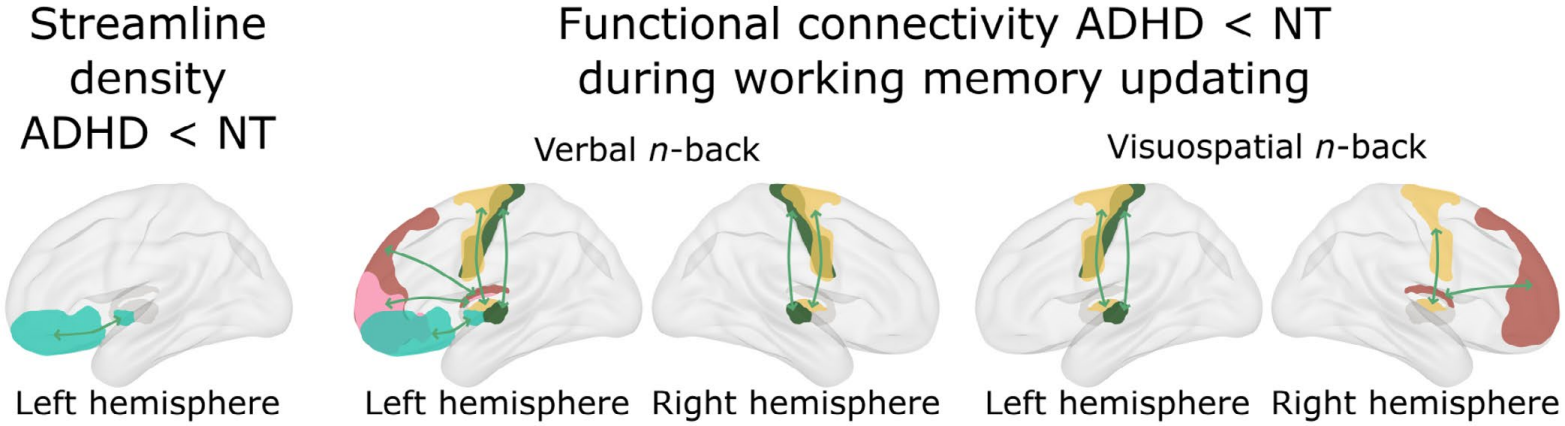
Overview of group differences in structural (left) and functional (right) connectivity. In the ADHD group, reduced streamline density was found between the left rostral putamen and orbitofrontal regions. Functional connectivity was decreased in adults with ADHD during working memory tasks, but not during resting state or the naturalistic attention task. During both verbal and visuospatial working memory, group differences emerged in motor and dlPFC circuits, with additional differences in ventrolateral PFC (vlPFC) and OFC circuits during the verbal task. See Section 3 for full details.

Overall, our findings outline ADHD‐related structural and functional alterations in the frontostriatal circuits. Disruptions in functional connectivity appear to be related to striatum‐dependent cognitive processes, such as working memory. Notably, the emergence of group differences during working memory processes, but not during rest or the naturalistic attention task, underscores the importance of selecting appropriate paradigms to map brain‐behaviour relationships, as task‐based settings may provide a more revealing analysis of these relationships in both non‐clinical and clinical populations (Finn 2021).

### 4.1 Individual Reconstruction of the Frontostriatal Circuits

Individualised definition of brain areas is crucial in neuroscience, as brain structure and functional patterns vary substantially across individuals (Gordon et al. 2017; Kong et al. 2021; Mueller et al. 2013). This is particularly relevant in disorders such as ADHD, which are underpinned by heterogenous variations in brain structure, function, and connectivity (Lei et al. 2014; Pan et al. 2025; Qian et al. 2019; Saad et al. 2017; Shang et al. 2018; Wolfers et al. 2020), driven by developmental changes shaped by genetic variability and environmental factors (Faraone et al. 2024). The inter‐individual variability of both symptoms and their biological correlates is a core challenge in ADHD research (Bu et al. 2025; Faraone et al. 2024; Firouzabadi et al. 2022). By selecting individually defined white matter tracts and the associated cerebro‐cortical ROIs, we aimed to mitigate the loss of true participant‐specific variability that could occur when using atlas‐based ROI selection.

While functional co‐fluctuations between brain regions depend on both direct and indirect structural connections, the relationship between structural and functional connectivity is complex (Fotiadis et al. 2024). Structural connectivity reflects the brain's long‐term architectural framework and tends to remain relatively stable over time. In contrast, functional connectivity is dynamic, context‐sensitive, and influenced by cognitive states and environmental demands. This divergence suggests a gradient in structure–function coupling across the brain: strong coupling is typically found in primary sensory and motor areas, whereas it becomes weaker in transmodal regions responsible for higher‐order processes such as attention, emotional regulation, and working memory (Fotiadis et al. 2024). The discrepancy between structural and functional connectivity was also apparent in the present study, showing only modest spatial overlap across frontostriatal circuits and task types. Similar modest structure–function coupling in frontostriatal pathways has been reported in macaques (Tang et al. 2025). Together, these findings underscore the need for further work on frontostriatal structure–function relationships across task types.

The structural connectivity patterns were topologically largely reproducible across participants, but the specific spatial location and size of each cerebro‐cortical endpoint region showed some individual variability which was slightly more pronounced in the ADHD group. Similar inter‐individual variability has been observed previously regarding both frontostriatal connectivity (Gordon et al. 2021; Tziortzi et al. 2014) and global functional organisation of the brain more generally (Gordon et al. 2017; Kong et al. 2021; Mueller et al. 2013). Comparing individually parcelled tracts with tracts conducted using pre‐selected cerebro‐cortical areas revealed that the individual tracts had denser connections (i.e., included more streamlines per voxel) to the frontal areas. This indicates that our individual tract formation approach was successful in pinpointing the most relevant cerebro‐cortical counterparts for different striatal regions. Individual connectivity disparities emphasise the need for more participant‐specific ways of describing brain function and structure rather than relying on atlas‐based brain parcellations. It has been proposed that mapping of individual connectomes is an important step forward in analysing the brain both in health and disease (Gordon et al. 2017). Furthermore, individualised, tractography‐based mapping could help refine targeting of relevant cerebro‐cortical areas for neuromodulation therapies used in psychiatric and neurological disorders (Lioumis et al. 2025; Ukharova et al. 2025). This approach may also facilitate improved detection, prognosis, and personalised treatment in these conditions, analogous to possibilities enabled by precision functional mapping (Demeter and Greene 2024). Our research suggests that DW‐MRI tractography can be utilised to define individual connectivity patterns for structural and functional analyses, at least in frontostriatal regions.

### 4.2 ADHD‐Related Differences in Frontostriatal White Matter

Our findings add to previous literature highlighting the centrality of OFC in ADHD symptomology (Norman et al. 2016; Yu et al. 2023), as we observed structural alterations in frontostriatal tracts originating from these regions. The present results align with previous reports of white matter abnormalities in striato‐OFC pathways, identified in both volumetric (Cupertino et al. 2020) and microstructural studies (Konrad et al. 2010; Schweren et al. 2016). The OFC plays a central role in regulating emotional and motivational behaviour (Rolls 2023), and aberrant OFC‐connectivity may underlie the atypical reward‐related decision making and emotional difficulties frequently reported in ADHD (Shaw et al. 2014). Supporting this view, individuals with ADHD have been shown to exhibit disrupted connectivity (Ceceli et al. 2020; Ma et al. 2016; Park et al. 2016) and altered activation patterns (see review by Rubia 2018) in the OFC and striatum during tasks involving motivational and emotional processing. Although the differences were observed only in the left hemisphere, there were no statistically significant group × hemisphere interactions. Overall, the structural aberrancies were susceptible to choices made in ROI formation, making them less robust, and must be interpreted accordingly.

Previous investigations of frontostriatal structural connectivity in adult ADHD are scarce. While previous evidence points to altered RD, AD, and FA in frontostriatal tracts in ADHD (Chaim et al. 2014; Chiang et al. 2022; Gehricke et al. 2017; Konrad et al. 2010; Schweren et al. 2016), no microstructural changes were observed in the present study in the main analyses. However, using more stringent thresholds in ROI selection we observed increased RD and AD in the ADHD group in tracts originating from orbitofrontal regions, but due to the complementary nature of these analyses the results are not discussed further. Regarding connectivity strength, some studies have reported both increased and decreased structural connectivity of striatal and frontal regions, but their network‐based analytical approaches do not allow us to examine frontostriatal connections specifically (Sidlauskaite et al. 2015; Tolonen et al. 2023; Wang et al. 2021). However, despite methodological differences, our findings are broadly consistent with those of the mega‐analysis by Cupertino et al. (2020), which reported reduced white matter volume in an OFC‐striatal component: Both results converge on the interpretation of diminished connectivity between orbitofrontal regions and the striatum in adult ADHD.

### 4.3 Aberrant Functional Connectivity During Working Memory Tasks

The present study sheds light on the neural basis of working memory deficits in ADHD (Alderson et al. 2013; Willcutt et al. 2005). While general frontostriatal circuit involvement was previously highlighted in neurocognitive models (Castellanos and Tannock 2002; Sonuga‐Barke 2002), our findings pinpoint precise pathways impacted. Both verbal and spatial working memory hypoactivation in ADHD appeared in somewhat similar frontostriatal circuits, including dlPFC, premotor, and motor regions. While the dlPFC's role in working memory deficits in ADHD is well established (Bédard et al. 2014; Cortese et al. 2012; Massat et al. 2012; McCarthy et al. 2014; Tolonen et al. 2024), our results indicate that altered connectivity between the dlPFC and striatum is a key mechanism behind these aberrations. The left dlPFC was affected during the verbal working memory task, along with the left vlPFC and OFC circuits, whereas the right dlPFC showed effects during the spatial task. This pattern accords with canonical hemispheric lateralisation of working memory domains (Nagel et al. 2013; Walter et al. 2003). However, no significant group × task × hemisphere interactions were detected in the present sample. Aberrant connectivity from the ventral caudate nucleus during the verbal task may relate to overlapping cerebro‐cortical representations with the dorsal caudate nucleus or the vlPFC's role in verbal working memory and related deficits observed in ADHD (Cortese et al. 2012; Rottschy et al. 2012).

Divergent connectivity in motor and premotor circuits, while perhaps unexpected, aligns with their supporting role in working memory processes (Marvel et al. 2019; Rottschy et al. 2012; Wang et al. 2019; Yaple et al. 2019; Yaple and Arsalidou 2018). Moreover, aberrations within the motor network have repeatedly been proposed as a key feature of ADHD (Cortese et al. 2012). Similarly, aberrant OFC connectivity might reflect its well‐documented involvement in ADHD (Norman et al. 2016; Yu et al. 2023), or its role in networks suppressed during tasks (Girn et al. 2024). Evidence also suggests that the OFC supports complex functions like maintaining the relative recency of presented stimuli (Barbey et al. 2011; Johnson et al. 2022). Collectively, these findings highlight that ADHD‐related working memory deficits involve not only canonical frontostriatal pathways but also altered contributions from motor and orbitofrontal circuits.

Despite connectivity findings, no differences in in‐scanner *n*‐back task accuracy were noted between the groups. However, the ADHD participants showed faster reaction times in many task levels, which might reflect their impulsive behaviour tendencies. Nevertheless, discrepancy between brain activation and behavioural findings in ADHD studies is not unique to our study (see e.g., Massat et al. 2012; Valera et al. 2005). The MRI environment might influence task engagement and performance (e.g., van Maanen et al. 2016), and potentially mask group differences that might appear elsewhere. Indeed, the adults with ADHD performed worse than the NT adults on the out‐of‐scanner spatial running memory task. However, we found no associations between running memory performance and functional connectivity during the spatial task, limiting interpretation of the group differences in functional connectivity. This dissociation can also result from the two task types likely engaging partially distinct cognitive processes (Bunting et al. 2006; Huang et al. 2025). Differences in functional connectivity may arise from factors not captured by hit rates or reaction times, such as engagement of additional brain systems reflecting alternative task strategies (Chauvin et al. 2021; Fassbender and Schweitzer 2006).

Contrary to a mega‐analysis on children with ADHD (Norman et al. 2024), we found no group differences in frontostriatal connectivity during resting state in adults. However, developmental changes in symptomatology and brain organisation (Franke et al. 2018; Grayson and Fair 2017; Lebel et al. 2008; Mills et al. 2016) complicate direct child‐adult comparisons. Though adult studies have noted altered striatal and frontal connectivity during rest (Hoekzema et al. 2013; Lin and Gau 2015; McCarthy et al. 2013; Sörös et al. 2019; Zhao et al. 2017), reports of disrupted connectivity between these regions specifically are scarce. The present results also align with our previous findings, showing differences in working memory‐related but not in resting‐state whole‐brain connectivity (Tolonen et al. 2024). Despite the growing popularity of resting‐state paradigms and their important discoveries of connectivity alterations in clinical populations, there are major difficulties in meaningfully interpreting their findings (Finn 2021). A focus on more relevant task designs could better reveal brain correlates related to symptom manifestation in everyday life.

However, differences between task and resting conditions do not explain the lack of group differences in the naturalistic attention task, which involves watching a film with distractors to mimic daily life attentional lapses (Salmi, Metwaly, et al. 2020). Our earlier work showed ADHD‐related desynchronisation in attention‐related frontal areas during this task (Salmi, Metwaly, et al. 2020), and other studies have similarly reported reduced frontal activation and aberrant connectivity during attentional processing in ADHD (Hart et al. 2013; Kowalczyk et al. 2022). Furthermore, in our prior work, recall accuracy after movie viewing correlated with coherence of brain activation in the inferior frontal gyrus among participants with ADHD and in the frontal eye fields among NT controls (Salmi, Metwaly, et al. 2020). However, no association between frontostriatal functional connectivity and recall accuracy was found in the present study. The role of frontostriatal circuits in this paradigm and other naturalistic tasks remains unclear due to limited research. Given that naturalistic tasks evoke strong neural responses alongside diverse emotional and cognitive processes (Sonkusare et al. 2019), the absence of detectable group differences here was unexpected. Nevertheless, the current findings underscore that the detectability of neural alterations in ADHD may be highly context dependent.

Group differences emerged during working memory tasks but not during the other functional conditions, suggesting that frontostriatal abnormalities may be most detectable when circuits are engaged by core operations such as working memory updating. Group × task interactions were observed in motor circuits implicated in motor planning and execution, suggesting potential differences in psychomotor processing between the adults with ADHD and NT controls, particularly given that these were the only tasks requiring overt motor responses. Notably, however, motor circuits also have a strong supporting role in working memory processes (Marvel et al. 2019; Rottschy et al. 2012; Wang et al. 2019; Yaple et al. 2019; Yaple and Arsalidou 2018). Taken together, our findings likely reflect task‐evoked, domain‐specific dysfunction related to working memory deficits (or psychomotor operations) rather than stable, trait‐like alterations that generalise across conditions.

### 4.4 Limitations

Several limitations of the present study should be acknowledged. The relatively modest sample size limited the statistical power of our analyses, particularly given the need to correct for multiple comparisons. Some effects may not have survived FDR correction with this sample size. It also precluded more detailed subgroup investigations, such as examining potential gender differences. In addition, because of the limited a priori information about the effect sizes of the studied phenomena, we did not conduct formal power calculations, which represents an additional limitation. Furthermore, in order to reduce the impact other clinical conditions might have on the results, individuals with comorbid psychiatric or neurological conditions were excluded from the present study. While this approach improved the specificity of the results to ADHD, it also reduced the generalisability of our findings, as the sample represents a more restricted subset of the broader ADHD population. A substantial proportion of the participants in the ADHD group were also receiving stimulant treatment, and consequently, the differences between the ADHD and NT participants cannot fully be separated from effects resulting from stimulant exposure. This also prevented adjusting for medication in group comparisons. In the within‐group analysis, stimulant use was associated with small effects that varied in direction, which makes the interpretation more complex. For functional connectivity, the association with stimulant use was negative and for structural connectivity it was positive. However, these findings should be viewed as provisional given the small (lis)dexamfetamine (*n* = 4) and non‐stimulant (*n* = 7) subgroups. In terms of imaging parameters, the DW‐MRI data were acquired using a traditional single‐shell, that is, single diffusion‐weighting, sequence in contrast to a more advanced multi‐shell acquisition, limiting the range of possible analysis methods that could be implemented, especially regarding microstructural features. The use of single‐shell, single‐tissue CSD may also have affected tractography accuracy because it can overestimate the proportion of white matter in voxels that also contain grey matter or CSF, potentially introducing spurious FOD peaks in those regions (Jeurissen et al. 2014). Furthermore, our complementary analyses indicated that the structural group differences were dependent on the chosen voxel‐wise threshold, suggesting limited robustness and sensitivity to subjective threshold selection. Lastly, our connectivity analyses focused exclusively on direct connections between striatal and cerebro‐cortical regions, which may have overlooked more complex topological alterations in connectivity patterns that could be revealed through more comprehensive analytical frameworks.

## 5 Conclusions

The present study provides significant insights into the complex landscape of structural and functional alterations in frontostriatal circuits associated with adult ADHD. Our individualised approach of frontostriatal connectivity delineation revealed inter‐individual variability in connectivity patterns, underscoring the limitations of atlas‐based methods and the need for personalised assessments in ADHD research. Furthermore, functional connectivity findings indicate that functional disruptions in frontostriatal circuits may be particularly linked to striatum‐dependent cognitive processes, notably working memory.

## Funding

This study received funding from the Research Council of Finland (grant numbers for M.L.: 260276 and 323251; for K.A.: 260054 and 297848; for J.S.: 325981 and 328954), Åbo Akademi University Endowment for the BrainTrain project (for M.L.), Finnish Cultural Foundation (for T.R.), and Vilho, Yrjö, and Kalle Väisälä Foundation of the Finnish Academy of Science and Letters (for T.T.).

## Ethics Statement

The study was reviewed by the Ethics Committee for Gynaecology and Obstetrics, Paediatrics, and Psychiatry of the Helsinki University Hospital.

## Consent

All participants gave written informed consent to participate in the study.

## Conflicts of Interest

The authors declare no conflicts of interest.

## Supporting information


**Data S1:** hbm70652‐sup‐0001‐cortical_ROI_maps_in_MNI.zip.

**Table S1:** Demographic and clinical characteristics of the participants.
**Table S2:** MANOVA results for the effect of group (ADHD/NT) on the multivariate pattern of FA, AD, and RD.
**Table S3:** Dice coefficients for the spatial overlap between strongest structural and functional connectivity voxels. Mean coefficients and standard deviations in parentheses.
**Figure S1:** Illustration of the cerebro‐cortical regions selected for atlas‐based analyses of structural connectivity. Atlas‐based analyses were only conducted for evaluating the robustness of individual tract reconstruction.
**Figure S2:** Differences in streamline density between individually defined cerebro‐cortical ROIs (blue) and pre‐defined atlas‐based ROIs (orange). Violin plots illustrating density distributions, individual data points, and boxplots are presented. Caud. nucl. = caudate nucleus, put. = putamen, L = left, *R* = right.
**Figure S3:** Group differences in the number of streamlines in each frontostriatal circuit. Violin plots illustrating density distributions, individual data points, and boxplots are presented. Caud. nucl. = caudate nucleus, put. = putamen, L = left, R = right.
**Figure S4:** Group differences in streamline density in each frontostriatal circuit. Violin plots illustrating density distributions, individual data points, and boxplots are presented. Caud. nucl. = caudate nucleus, put. = putamen, L = left, R = right.
**Figure S5:** Group differences in functional connectivity during digit *n*‐back task in each frontostriatal circuit. Violin plots illustrating density distributions, individual data points, and boxplots are presented. Caud. nucl. = caudate nucleus, put. = putamen, L = left, R = right.
**Figure S6**. Group differences in functional connectivity during visuospatial *n*‐back task in each frontostriatal circuit. Violin plots illustrating density distributions, individual data points, and boxplots are presented. Caud. nucl. = caudate nucleus, put. = putamen, L = left, R = right.
**Figure S7:** Group differences in functional connectivity during resting state in each frontostriatal circuit. Violin plots illustrating density distributions, individual data points, and boxplots are presented. Caud. nucl. = caudate nucleus, put. = putamen, L = left, R = right.
**Figure S8:** Group differences in functional connectivity during the naturalistic attention task in each frontostriatal circuit. Violin plots illustrating density distributions, individual data points, and boxplots are presented. Caud. nucl. = caudate nucleus, put. = putamen, L = left, R = right.

## Data Availability

The data that support the findings of this study are available on request from the corresponding author. The data are not publicly available due to privacy or ethical restrictions.
