# Supplementary material for: Individualised Structural and Functional Frontostriatal Connectivity Mapping in Adult ADHD
Source: Hum Brain Mapp. 2026 Oct 1;47(14):e70652. doi: 10.1002/hbm.70652 (PMC13631470; doi:10.1002/hbm.70652)
Supplement: Supplementary file 1 [file HBM-47-e70652-s002.docx]

Supplementary results

**Article title**

Individualised structural and functional frontostriatal connectivity mapping in adult ADHD

**Authors**

Tuija Tolonen, Matti Laine, Kimmo Alho, Sami Leppämäki, Pekka Tani, Anniina Koski, Juha Salmi, Timo Roine

**Supplementary Tables**	pp. 2–5

**Supplementary Figures**	pp. 6–13

**Complementary analyses results**	pp. 14

| **Supplementary Table 1.** *Demographic and clinical characteristics of the participants.* | | | | |
| --- | --- | --- | --- | --- |
| Characteristics | ADHD  (*n* = 40) | NT  (*n* = 36) | Test statistic (*df*) | *p* |
| Age, *M (SD)* | 28.35 (5.13) | 28.42 (7.81) | *t* (59.38) *=* 0.04 | .97 |
| Gender, male/female | 17/23 | 14/22 | χ² (1) = 0.10 | .75 |
| Handedness, right/left | 34/6 | 33/3 | χ² (1) = 0.81 | .37 |
| General cognitive abilities, *M (SD)* |  |  |  |  |
| WAIS-III Vocabulary | 11.38 (2.54) | 11.11 (2.44) | *t* (74) = -0.46 | .65 |
| WAIS-III Matrix Reasoning | 12.43 (2.82) | 13.17 (2.22) | *t* (74) = 1.26 | .21 |
| Education* | 6/11/7/1/5/5 | 3/12/2/1/5/9 | χ² (5) = 4.84 | .44 |
| Mood, *M (SD)* | 5.15 (3.42) | 5.00 (3.67) | *t* (73) = -0.18 | .86 |
| Alcohol consumption, *M (SD)* | 4.30 (2.39) | 3.57 (2.03) | *t* (73) = -1.41 | .16 |
| ADHD symptoms (ASRS), *M (SD*) |  |  |  |  |
| Inattention | 23.36 (5.08) | 12.78 (5.54) | *t* (74) = -8.69 | < .001 |
| Hyperactivity-impulsivity | 19.58 (7.27) | 10.19 (4.96) | *t* (69.15) = -6.63 | < .001 |
| Naturalistic attention task post-quiz performance, *M (SD)* | 21.50 (4.21) | 19.36 (6.15) | *t* (74) = -1.79 | .08 |
| Working memory performance, *M (SD*) |  |  |  |  |
| WAIS-III Digit Span | 11.50 (2.86) | 11.97 (3.06) | *t* (74) = 0.69 | .49 |
| Verbal running memory | 3.37 (1.84) | 3.53 (1.66) | *t* (70) = 0.39 | .70 |
| Spatial running memory | 3.31 (2.13) | 4.26 (1.81) | *t* (71) = 2.05 | .04 |
| Visuospatial 1-back, hit rate (%) | 88.45 (18.60) | 92.67 (6.95) | *t* (74) = 1.28 | .20 |
| Visuospatial 2-back, hit rate (%) | 85.75 (19.56) | 85.78 (11.24) | *t* (74) = 0.01 | .99 |
| Visuospatial 3-back, hit rate (%) | 12.30 (11.05) | 10.33 (7.02) | *t* (74) = -0.91 | .36 |
| Digit 1-back, hit rate (%) | 90.35 (19.39) | 93.83 (5.07) | *t* (74) = 1.05 | .30 |
| Digit 2-back, hit rate (%) | 86.10 (19.38) | 86.56 (10.79) | *t* (74) = 0.13 | .90 |
| Digit 3-back, hit rate (%) | 14.60 (9.81) | 12.63 (6.94) | *t* (74) = -1.04 | .30 |
| Visuospatial 1-back, RT (ms) | 661 (149) | 763 (161) | *t* (74) = 2.85 | .006 |
| Visuospatial 2-back, RT (ms) | 832 (215) | 945 (209) | *t* (74) = 2.31 | .02 |
| Visuospatial 3-back, RT (ms) | 877 (220) | 1023 (220) | *t* (74) = 2.89 | .005 |
| Digit 1-back, RT (ms) | 662 (158) | 715 (156) | *t* (74) = 1.45 | .15 |
| Digit 2-back, RT (ms) | 845 (222) | 952 (215) | *t* (74) = 2.12 | .04 |
| Digit 3-back, RT (ms) | 901 (240) | 1010 (235) | *t* (74) = 1.98 | .05 |
| *Note. df* = degrees of freedom, *M* = mean, *SD* = standard deviation, ASRS = Adult ADHD Self-Report Scale, RT = reaction time, ms = millisecond. *Education scale in order of appearance: comprehensive school / upper secondary school / vocational school / community college level / bachelor’s degree / master’s degree. | | | | |

| **Supplementary Table 2.** *MANOVA results for the effect of group (ADHD/NT) on the multivariate pattern of FA, AD, and RD.* | | | |
| --- | --- | --- | --- |
| Frontostriatal circuit | Wilk’s Λ | *F* (*df1, df2*) | *p* |
| Dorsal caudate nucleus L | .963 | 0.87 (3, 68) | .462 |
| Dorsal caudate nucleus R | .958 | 1.05 (3, 71) | .378 |
| Ventral caudate nucleus L | .940 | 1.48 (3, 69) | .228 |
| Ventral caudate nucleus R | .988 | 0.29 (3, 69) | .834 |
| Rostral caudate nucleus and nucleus accumbens L | .859 | 3.84 (3, 70) | .013 |
| Rostral caudate nucleus and nucleus accumbens R | .836 | 4.58 (3, 70) | .006 |
| Caudal putamen L | .973 | 0.64 (3, 69) | .595 |
| Caudal putamen R | .935 | 1.56 (3, 67) | .207 |
| Dorsal putamen L | .888 | 2.90 (3, 69) | .041 |
| Dorsal putamen R | .973 | 0.62 (3, 67) | .605 |
| Rostral putamen L | .933 | 1.70 (3, 71) | .175 |
| Rostral putamen R | .872 | 3.38 (3, 69) | .023 |
| *Note. df* = degrees of freedom, L = left, R = right. | | | |

| **Supplementary Table 3.** *Dice coefficients for the spatial overlap between strongest structural and functional connectivity voxels. Mean coefficients and standard deviations in parentheses.* | | | | |
| --- | --- | --- | --- | --- |
| Task  Frontostriatal circuit | Resting state | Naturalistic attention task | Digit *n*-back | Visuospatial *n*-back |
| Dorsal caudate nucleus L | 0.23 (0.03) | 0.23 (0.03) | 0.24 (0.03) | 0.24 (0.03) |
| Dorsal caudate nucleus R | 0.23 (0.03) | 0.23 (0.03) | 0.24 (0.03) | 0.23 (0.03) |
| Ventral caudate nucleus L | 0.23 (0.03) | 0.24 (0.03) | 0.23 (0.03) | 0.23 (0.03) |
| Ventral caudate nucleus R | 0.24 (0.03) | 0.24 (0.03) | 0.24 (0.03) | 0.24 (0.03) |
| Rostral caudate nucleus and nucleus accumbens L | 0.17 (0.04) | 0.17 (0.04) | 0.17 (0.04) | 0.17 (0.04) |
| Rostral caudate nucleus and nucleus accumbens R | 0.16 (0.04) | 0.17 (0.04) | 0.16 (0.05) | 0.16 (0.04) |
| Caudal putamen L | 0.19 (0.03) | 0.20 (0.04) | 0.19 (0.03) | 0.19 (0.03) |
| Caudal putamen R | 0.20 (0.03) | 0.20 (0.04) | 0.20 (0.04) | 0.19 (0.04) |
| Dorsal putamen L | 0.21 (0.03) | 0.21 (0.03) | 0.22 (0.03) | 0.21 (0.03) |
| Dorsal putamen R | 0.21 (0.03) | 0.22 (0.03) | 0.22 (0.03) | 0.22 (0.03) |
| Rostral putamen L | 0.19 (0.04) | 0.19 (0.04) | 0.19 (0.04) | 0.19 (0.04) |
| Rostral putamen R | 0.17 (0.05) | 0.17 (0.05) | 0.17 (0.05) | 0.17 (0.05) |
| *Note. df* = degrees of freedom, L = left, R = right. | | | | |

**Supplementary Figure 1**. Illustration of the cerebro-cortical regions selected for atlas-based analyses of structural connectivity. Atlas-based analyses were only conducted for evaluating the robustness of individual tract reconstruction.

**Supplementary Figure 2.** Differences in streamline density between individually defined cerebro-cortical ROIs (blue) and pre-defined atlas-based ROIs (orange). Violin plots illustrating density distributions, individual data points, and boxplots are presented. Caud. nucl. = caudate nucleus, put. = putamen, L = left, R = right.

**Supplementary Figure 3.** Group differences in the number of streamlines in each frontostriatal circuit. Violin plots illustrating density distributions, individual data points, and boxplots are presented. Caud. nucl. = caudate nucleus, put. = putamen, L = left, R = right.

**Supplementary Figure 4.** Group differences in streamline density in each frontostriatal circuit. Violin plots illustrating density distributions, individual data points, and boxplots are presented. Caud. nucl. = caudate nucleus, put. = putamen, L = left, R = right.

**Supplementary Figure 5.** Group differences in functional connectivity during digit *n*-back task in each frontostriatal circuit. Violin plots illustrating density distributions, individual data points, and boxplots are presented. Caud. nucl. = caudate nucleus, put. = putamen, L = left, R = right.

**Supplementary Figure 6.** Group differences in functional connectivity during visuospatial *n*-back task in each frontostriatal circuit. Violin plots illustrating density distributions, individual data points, and boxplots are presented. Caud. nucl. = caudate nucleus, put. = putamen, L = left, R = right.

**Supplementary Figure 7.** Group differences in functional connectivity during resting state in each frontostriatal circuit. Violin plots illustrating density distributions, individual data points, and boxplots are presented. Caud. nucl. = caudate nucleus, put. = putamen, L = left, R = right.

**Supplementary Figure 8.** Group differences in functional connectivity during the naturalistic attention task in each frontostriatal circuit. Violin plots illustrating density distributions, individual data points, and boxplots are presented. Caud. nucl. = caudate nucleus, put. = putamen, L = left, R = right.

**Complementary analyses**

In the microstructural analyses, the main results were repeated (i.e. no group differences surviving FDR correction were observed) with thresholds 75/7500, 75/10000, and 100/7500. Using thresholds 125/7500 and 125/10000, MANOVA effects were found in the circuits seeded from the rostral caudate nucleus and nucleus accumbens bilaterally (the statistics are provided for thresholds 125/1000; left: *F*(3, 70) = 4.46, *p* = .006, Wilk's Λ = .840; right: *F*(3, 70) = 4.71, *p* = .005, Wilk's Λ = .832; both significant after FDR-correction). Post-hoc analyses revealed that in the tract seeded from left rostral caudate nucleus and nucleus accumbens the ADHD group had higher RD than the NT group (*t*(72) = 2.75, t = .008). In the tract seeded from the right rostral caudate nucleus and nucleus accumbens, the ADHD group had higher AD (*t*(72) = 2.68, *p* = .009) than the NT group. No effects were found for number of streamlines or streamline density.

In the functional connectivity analyses, there were slight changes to the specific circuits that were affected by ADHD when examining connectivity with different voxel- and clusterwise thresholds, compared with the main results presented in the main article. Specifically, in the verbal *n*-back task, circuits associated with left dorsal caudal nucleus were not significant with thresholds 75/7500, 75/10000, 125/7500, and 125/10000; instead, circuits associated with the right dorsal caudate nucleus became significant with thresholds 75/7500, 75/10000, and 100/7500. In the visuospatial *n*-back task, circuits associated with the right dorsal caudate nucleus and the right dorsal putamen were not significant with threshold 125/7500, and circuits associated with the right caudal putamen became significant with a threshold 125/10000. There were no other changes in functional connectivity with different thresholds.
